# Supplementary figures and images for: Scrub Typhus and Influenza A Co-Infection: A Case Report
Source: Pathogens. 2025 Jan 13;14(1):64. doi: 10.3390/pathogens14010064 (PMC11768316; doi:10.3390/pathogens14010064)

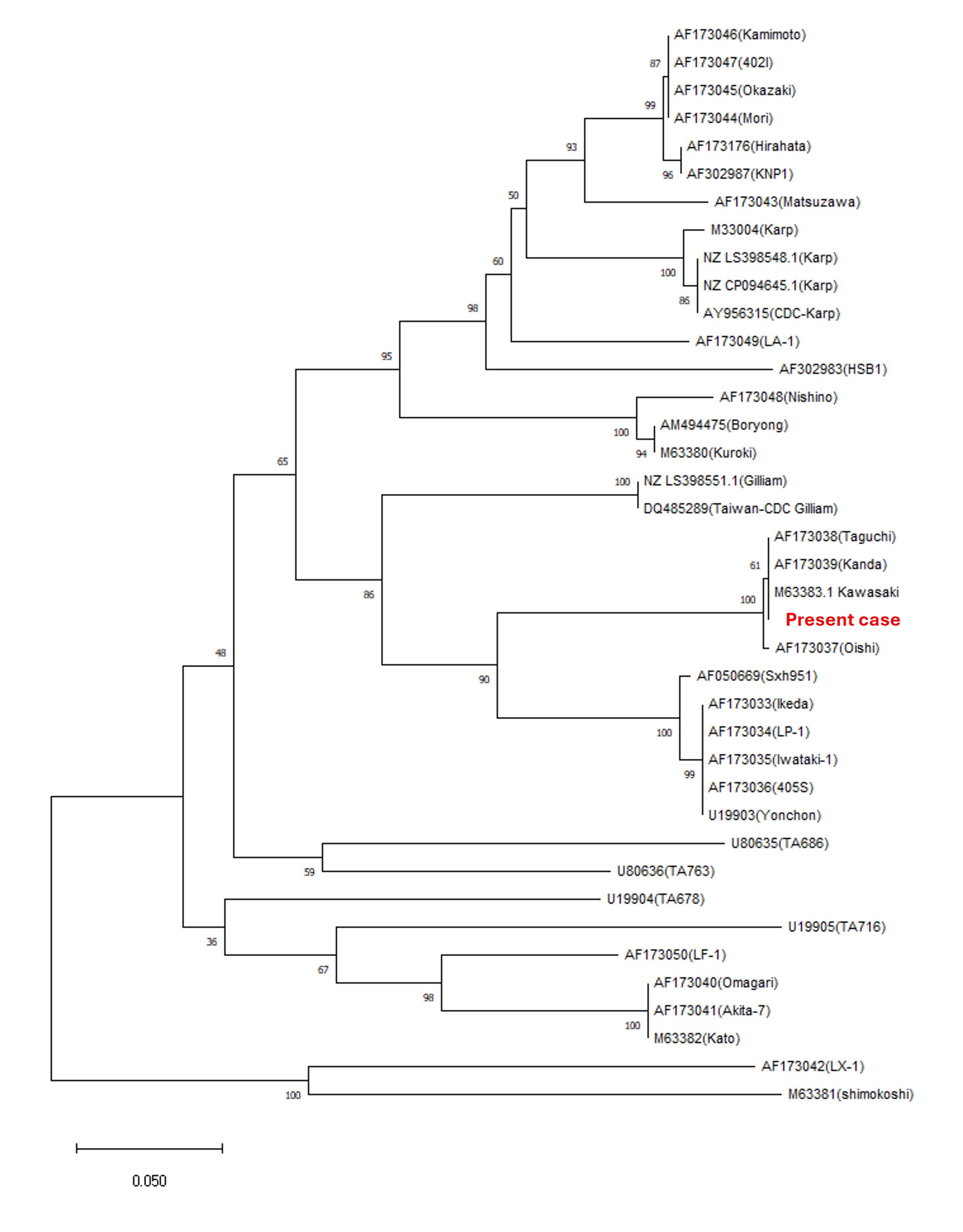

Supplement: Supplementary file 1 [file pathogens-14-00064-s001.zip › pathogens-3416922-supplementary/Supplementary Figure and Table/Supplementary Figure 1.jpg]
